# SAHCS 2021 Conference Summary

**DOI:** 10.4102/sajhivmed.v23i1.1371

**Published:** 2022-04-14

**Authors:** David C. Spencer

**Affiliations:** 1 Division of Infectious Diseases, Faculty of Medicine, University of the Witwatersrand, Johannesburg, South Africa; 2 Southern African HIV Clinicians Society, Johannesburg, South Africa

In a letter to the Lancet in 2017, Brian Williams and Reuben Granich remark (correctly) that ending AIDS by 2030 does not mean ‘the end of HIV’.^1^ Without a cure and an effective vaccine, an ever-expanding population of those living with the virus will require antiretroviral treatment (ART) indefinitely. So far, the world has no cure nor effective vaccine against HIV. However, carrying out something more to dramatically reduce the impact of virus on Africa’s current and future generations makes scientific and humanitarian sense. In October 2021, the Southern African HIV Clinicians Society (SAHCS) hosted its biannual 4-day conference in Johannesburg, South Africa (SA), with its opening plenary theme being ‘Game-changers for Epidemic Control’.

**FIGURE 1:**
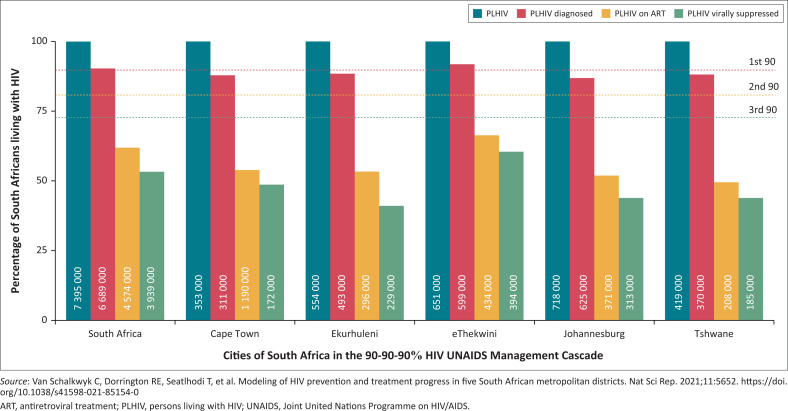
The 90-90-90%-HIV treatment cascade in South Africa and five of its metropolitan districts in 2018.

In 2014, the Joint United Nations Programme on HIV/AIDS (UNAIDS) launched an initiative to reduce HIV infections by 2020.^2^ Implementation of the 90-90-90% targets by 2020 began the following year. Unfortunately, few southern African countries met the 2020 deadline^3^ (Figure 1^4^); Botswana,^5^ a small country with a large HIV burden, met this deadline. Nevertheless, it is on track to achieve the revised 2030 targets by 2025: *95%* tested or diagnosed, *95%* (of these) on ART and *95%* (of these) virally suppressed. Can southern Africa end new HIV infections by 2030? Perhaps. However, it will require (1) a greater determination and commitment than previously observed, (2) an in-depth reappraisal of local targets, (3) settup of monitoring systems yearly or more frequently, (4) community mobilisation or input – to give momentum to the goal, and (5) a conscious effort to find the missing ‘KEY GROUPS’ that include:

- *the infected* but untreated
- *the uninfected* but at-risk
- *the treated* but unsuppressed or lost to care.

Will the point-of-care tests and the new antiretrovirals – the integrase inhibitors, the long-acting injectables, the new drug classes and the two-drug regimens – detail discussed at the October 2021 conference – achieve the 95-95-95% targets? Not on their own. Are the important scientific advances also discussed at the conference likely to induce citizens to arrive, get tested, take treatment and stay in care? No. Something more is needed.

We have learned much from the coronavirus disease 2019 (COVID-19) pandemic. What works? Clearly reported and accessible science (targeting *everyone*), truthful and frequent communication in national media by *credible scientific leaders*, defining a role for *everyone,* and the engagement of political and community leaders to mobilise constituencies towards an AIDS-free future.

**FIGURE 2:**
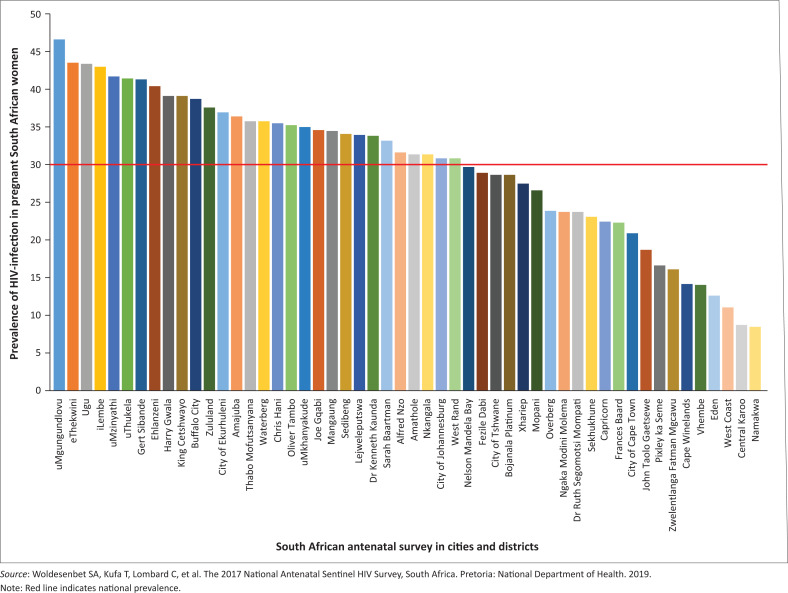
The prevalence of HIV among pregnant women by district in South Africa, the South African Antenatal Survey, 2017.

We have made mistakes. In aiming at the 2020 targets, children lagged behind, particularly the very young and adolescents,^6^ and also young women. The seroprevalence range of antenatal HIV in Soweto, South Africa, is currently at 27% – 30%. It has been at this level for the past two decades.^7^ Similar levels are reported in other South African districts (Figure 2).^8^

Jeremy Nel (chief of Infectious Diseases at the Helen Joseph Hospital, the University of the Witwatersrand, Johannesburg) chaired proceedings. The opening plenaries began with the ‘Game-Changers’: the *long-acting injectables* (Sinead Delaney-Moretlwe), *pre-exposure prophylaxis* (*PrEP*) *on demand for all* (Linda-Gail Bekker), *pharmacy-initiated antiretroviral treatment* (*PIMART*) (Francois Venter) and *U* (*undetectable*) = *U* (*untransmissible*) (Mandisa Dukashe). Each presentation covered new territory or developments in care. Pharmacy-initiated antiretroviral treatment is a ‘new’ venture that asks our community pharmacists to get involved. A decade ago, nurses were trained to assist with HIV-patient care – the nurse-initiated management of antiretroviral treatment (NIMART). Without the support of these nurses, it would have been impossible to roll out care to the near six million South Africans currently on treatment. A shortfall of approximately two million remain infected but untreated. Pharmacy-initiated management of antiretroviral treatment is an international movement, where community pharmacists assist clients with HIV testing, provide limited but focussed counselling and initiate the client onto HIV prevention medicines (PreP or PEP) or ART as is needed. This opens a portal for the untested and untreated to access ongoing HIV care. Pharmacy-initiated antiretroviral treatment is structured around a referral pathway from a pharmacist to clinician and/or HIV clinic. Similarly, the ‘Game-Changer’ concepts of PrEP, the long-acting injectables and the community U = U initiatives create novel opportunities that link ordinary people to game-changing programmes like PIMART.

In the opening plenary, I reviewed the history of HIV in Africa. A 100 years ago, circa 1884–1920, a simian immunodeficiency virus (SIVcpz) crossed from our primate relative, the chimpanzee, into humans in the forests of the Congo, Central Africa. The novelist and sea captain, Joseph Conrad who at that time worked the waters of the Congo River in search of rubber and ivory, describes the appalling living conditions of Congolese workers in his novel ‘The Heart of Darkness’. Nuno Faria, Paul Sharp, Beatrice Hahn, Michael Worobey and others have explored the origins of HIV in numerous articles over the last 30 years.^9,10,11^ While specific detail has been lost, Bayesian mathematics and phylogenetic mapping of HIV-DNA retrieved from the infected human tissue between 1959 and the present lay bare the virus’s evolution in humankind. AIDS first came to the attention of clinicians and scientists in 1981. Reports in the *New England Journal of Medicine (NEJM)* and *Morbidity and Mortality Weekly Report (MMWR)* described life-threatening opportunistic diseases in young American men and their sexual partners.^12,13^ But:

[*S*]hortly after the first reports of AIDS in the United States (US) in 1981, and the isolation of the virus (HIV-1) two years later, the disease was found to be established in the heterosexual populations of central and eastern Africa suggesting a much older and at that point, *a hidden history* of the pandemic in Africa.^9^ [*authors own italics*].

**FIGURE 3:**
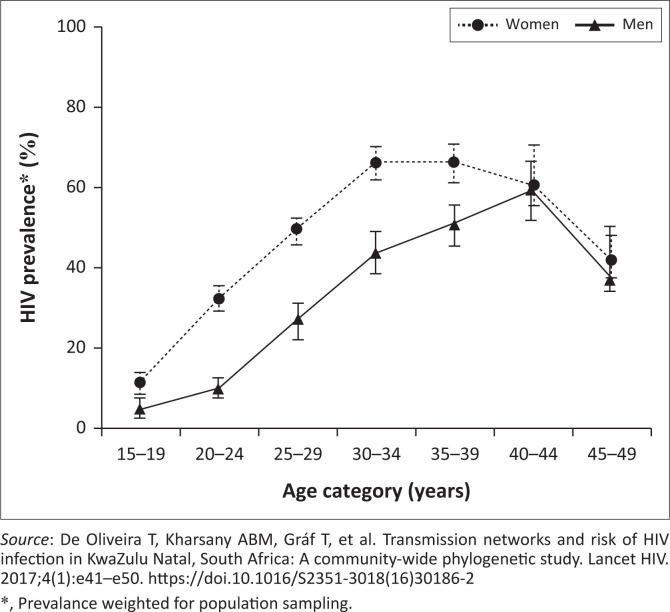
The HIV prevalence rates in a KwaZulu-Natal community in South Africa by age and gender.

Tulio de Oliveira’s description in 2017 of HIV transmission reported in the province of KwaZulu-Natal (KZN), SA, is a classic (Figure 3).^14^ The authors describe a cycle of sexual networking and viral transmission between adjacent rural and urban districts and between men and women in KZN. Women are particularly vulnerable to acquiring infection. The region is easily accessed by road and not far from the province’s commercial hub, eThekwini (Durban). Of the 8912 people enrolled in this study, 3969 (45%) tested HIV-positive. Some were already on treatment. However, a large number had detectable viral loads that permitted phylogenetic analysis and identification. In the under 25-year age group, the prevalence rates of HIV amongst the men and women were 7.6% (*n* = 1472), and 22.3% (*n* = 2224), respectively. The prevalence of HIV amongst those aged 25–40 years was high for both women (*n* = 2835, 59.8%) and men (*n* = 1548, 40.3%) but greater amongst women. The prevalence of HIV in women in both age groups outstripped that of men. At a time of sexual debut, girls or young women in this community had a three-fold greater risk of testing HIV positive than men of the same age. The researchers were able to fingerprint (‘cluster genotype’) viruses and follow their movement between groups. HIV transmission followed a path from the 25–40-year-old men to the younger, < 25 years women. Simultaneously, the virus spread horizontally between the men and women in the 25–40-year-old group. Women in the latter group also had a disproportionately higher level of infection. The story is not new:^15,16,17^

The differential status of men and women in almost every society is perhaps the most pervasive and entrenched inequity. Indeed, the feminisation of the AIDS epidemic in southern Africa clearly indicates the lack of power of women to participate as equals in the social freedoms of men.^18^

If the virus could speak and if we had ears to hear, it would repeat these words and ask why (our) society has performed so little to change this inequity?

Antiretroviral treatment programmes across southern Africa have been very successful. Numerically and geographically, SA is the largest of these. AIDS-related deaths have fallen. However, new infections contribute to the high prevalence rate. In her two talks on PrEP, Linda-Gail Bekker (Cape Town, SA) observed that this form of protection is poorly marketed and inadequately utilised in SA. Young women of schoolgoing age and students need protection. Pharmacy-initiated antiretroviral treatment would be an ideal pathway to do this. There will not be an end of new infections without addressing the gender inequity of HIV.

The conference was held virtually and broadcasted live from Johannesburg. The first day included the familiar format of workshops – new guidelines and old themes – the resistance workshop, how to write a scientific paper, and sponsored sessions: HIV and viral hepatitis, 5-flucytosine, cryptococcal meningitis (the AMBITION Study), and long-acting antiretroviral therapy. There were many familiar names amongst the speakers, and then the gender-affirming healthcare guidelines. I had read these some months earlier.^19^ For some, the guideline may appear to be a novelty. However, they do need to be taken seriously: they represent a ‘HEADS-UP’ to us all. What did Africa learn from the Stonewall riots of the 1960s? Or the gay men’s marches in Los Angeles and New York in the 1980s? Or the beautifully (and lovingly) crafted AIDS remembrance quilts for the thousands of young men who died from AIDS? It is time to allow individuality to blossom, to let people be who they are. *Ubuntu* is a local term meaning we ‘love one another’. The freedom of the LGBTQIA+ community was not gained without cost. The history of the epidemic in Africa is not *only* that of heterosexuals in need. But of gay and bisexual, of transgender men and women imprisoned in Uganda, murdered in SA, despised and denigrated by African politicians and Africa’s religious leaders … Take a look at these guidelines. Our world is changing. Be part of the new world. Learn the vocabulary. Congratulations to the conference organisers for placing a sensitive yet timely subject before the delegates.

Two presentations on the second day, in particular, caught my attention. ‘Is it possible to eliminate COVID-19: Lessons for and from HIV?’: Monica Gandhi (CA, US) and Shabir Mahdi (Johannesburg, SA) answered the question to the delight of the audience. Glenda Gray, chief of SAs Medical Research Council, chaired the session. The speakers had anticipated the global Omicron pandemic. They addressed the ethics of vaccine access in low-income countries, the waning of antiviral immune responses and the need for booster doses. Both presentations were a formidable display of speaker skills and the careful marshalling of scientific evidence. Definitely worth watching a second time! For those wanting more, I recommend a special report on COVID-19 in the 08 July 2021 edition of the NEJM. This is down-to-earth, ‘nuts and bolts’, science. Extremely helpful.^20^

The other presentation was Nicholas Paton’s (United Kingdom [UK]/Singapore) NADIA trial. This is a randomised, two-by-two factorial, open-label, non-inferiority trial of second-line ART in *n* = 464 patients from seven sub-Saharan African sites. The study was published in the NEJM in 2021. It makes for great reading.^21^ The design of the study allowed the authors to compare dolutegravir (DTG) with ritonavir boosted-darunavir following first-line NNRTI failure. At the same time, the second-line ART choice allowed continuation with the failing first-line tenofovir whilst comparing this with the traditional second-line choice of twice daily zidovudine. The results of viral genotyping from the start of second-line ART were only revealed at the 48-week assessment. In their NEJM paper, the authors conclude:

Dolutegravir in combination with NRTIs was effective in treating patients with HIV-1 infection, including those with extensive NRTI- resistance in whom *no NRTIs were predicted to have activity*. Tenofovir was non-inferior to zidovudine as second-line therapy.^21^ (p. 330)

It is a remarkable study. And it was a privilege to hear Nick Paton discuss it and interact with the session’s chair, Gary Maartens. Would this trial have been allowed in Europe and the US? What would the ethics committees have said? Perhaps not. However, it has taught me a great deal. Several years back another of Nick Paton’s African studies, the EARNEST trial, provided unexpected and thought-provoking results too.^22,23^ So do we throw away resistance testing? No. However, EARNEST and NADIA reveal that other factors are at play in ARV resistance, and that these fascinating studies are an essential part to understanding the big picture of HIV management.

Day 3 provided attendees with new data on viral load assessment from Annemarie Wensing and Lucas Hermans (the Netherlands) and the ‘Future of ART’ with Roy Gulick (US). Regarding ART’s future, there are multiple new options on the horizon, all of which are welcome. However, in Africa, cost and availability will be the driver. All three were state-of-the-art talks. In an early evening plenary on Day 3, Graeme Moyle (UK) provided perspective on weight gain in patients on DTG. Francois Venter’s ADVANCE study had pointed to weight problems with DTG and alafenamide (TAF). In brief, Prof. Moyle observed that weight change in patients living with HIV is influenced by their baseline health, and by innate differences in the antiretrovirals and the regimens used. Efavirenz appears to have protected against excessive weight gain. Alafenamide is associated with an approximately 2 kg initial increase in weight that is not lost by changing back to tenofovir – the TANGO and DOLAM studies. Women, and particularly women of African ancestry, may be at greater risk. An interesting talk that signals that we are not finished with the metabolic complications of ART. Many of us recall using stavudine, didanosine and indinavir in the years before contemporary ART was born. Prof. Moyle has an eye for unpacking the detail in the data. His discussion of the relevant studies was flawless.

The morning of day 4, 23 October, focussed on the Prevention of Mother to Child Transmission, Climate Change and HIV, the digitisation of the 2020 HIV Clinicians Society ART guidelines, Solid Organ Transplantation guidelines – an African first, the Clinicians Society’s HIV and Harm-Reduction guidelines – possibly another (local) first, and a late morning session on ‘Advanced HIV Disease’. The final session of the conference was in the afternoon. This saw Doug Richman (US), Mark Cotton (Stellenbosch, SA) and Mike Cohen (US) discuss ‘Cure’ (what I guess all of us had been waiting for since the beginning of the conference). They did not disappoint. Seeing and hearing Doug Richman took me back 20 years to when he and colleagues who are no longer with us, Charlie van der Horst, Dave Cooper, Mark Wainberg and Joop Lange, would visit African colleagues with advice and support. They were difficult years – a new epidemic, many AIDS deaths, toxic drugs, a denialist South African government and battles on many fronts. But much that is good has happened since.

The Southern African HIV Clinicians October 2021 conference was memorable. It rekindled the quest for a future without HIV. The organisers thank all participants, particularly, the speakers who freely and readily shared their time, knowledge and insights – many from remote corners of the globe! Can southern Africa look to a future that is free of HIV and AIDS? Yes, provided that there is commitment from all of us.
